# Dynamic trajectory of platelet counts after the first cycle of induction chemotherapy in AML patients

**DOI:** 10.1186/s12885-022-09601-5

**Published:** 2022-05-01

**Authors:** Yazhen Bi, Zhaohui Wang, Saran Feng, Yan Wang, Yang Zhao, Hong Li, Jingyi Yu, Qian Liu, Chuansheng Zhu, Mingzhuo Li

**Affiliations:** 1 Shandong Qianfoshan Hospital, Cheeloo College of Medicine, Shandong University, Jinan, Shandong China; 2 Shandong Provincial Qianfoshan Hospital, Shandong University, Jinan, Shandong China; 3 Department of Hematology, Haici Medical Group Qingdao, Qingdao, Shandong China; 4 Department of Biostatistics, School of Public Health, Nanjing Medical University, Nanjing, Jiangsu China; 5 Center for Big Data Research in Health and Medicine, The First Affiliated Hospital of Shandong First Medical University & Shandong Provincial Qianfoshan Hospital, Jinan, Shandong China

**Keywords:** Acute myeloid leukemia, Trajectory, Platelet counts, All-cause mortality

## Abstract

**Background:**

Platelet counts varied over time after induction chemotherapy. We aimed to investigate the different trajectories of platelet counts after the first cycle of induction chemotherapy in patients newly diagnosed with acute myeloid leukemia.

**Methods and results:**

In total, 149 individuals were included in this study. We identified four distinct trajectories using a group-based trajectory model: low- stability group (*n* = 27, 18.12%), low-level decrease–medium elevation group (*n* = 42, 28.19%), low-level decrease–high elevation group (*n* = 60, 40.27%), and high-level decrease–medium elevation group (*n* = 20, 13.42%). The baseline characteristics of the high-level decrease–medium elevation group included higher platelet count, lower white blood cell count, lower percentage of bone marrow blasts, and lower rates of complete remission after the first cycle of induction chemotherapy. Compared with the low-stability group, the hazard ratios were 0.32 (95% confidence interval, 0.15–0.68) for the low-level decrease–medium elevation group, 0.31 (95% confidence interval, 0.15–0.63) for the low-level decrease–high elevation group, and 0.35 (95% confidence interval, 0.13–0.89) for the high-level decrease–medium elevation group after adjustment for age and gender by Cox proportional hazard regression. Compared with the low-stability group, the hazard ratios were 0.33 (95% confidence interval, 0.14–0.77) for the low-level decrease–medium elevation group and 0.31 (95% confidence interval, 0.14–0.67) for the low-level decrease–high elevation group after adjustment for age, gender, white blood cell count, and bone marrow blasts. These associations persisted after adjusting for age, gender, white blood cell count, bone marrow blasts, and platelet count.

**Conclusion:**

The dynamic trajectory of platelet counts after the first cycle of induction chemotherapy is a significant predictor of all-cause mortality in patients with acute myeloid leukemia. Timely intervention should be considered for the low-stability group. The low-level decrease–medium elevation and low-level decrease-high elevation groups were independent protective factors for all-cause mortality.

## Background

Acute myeloid leukemia (AML) is a heterogeneous disease with variations in its clinical features, cytogenetics, molecular abnormalities, and epigenetic alterations. The clinical outcome of patients with AML varies widely, from survival of a few days to remission [1]. Therefore, precise diagnosis and risk stratification are crucial [2, 3]. Currently, the selection of appropriate therapeutic methods for AML mainly depends on the age of the patient and risk stratification, including cytogenetic and molecular features [4]. Nevertheless, the data collected after the initiation of AML treatment can potentially be as useful as the pretreatment characteristics [5]. Therefore, post-treatment data may be used in developing treatment decision-making strategies [6]. In 2003, an international working group proposed a criterion for classifying treatment responses: complete remission (CR) for incomplete blood count recovery (CRi) [7]. One subclass of the CRi criteria is CRp. CRp means that the patient fulfills all the criteria except for a platelet (PLT) count of < 100,000/L. Roland B et al. used data from 6,283 patients to indicate that patients who achieved CR had a greater chance of living beyond 3 or 5 years than those who achieved CRp [8]. CRp is significant for the prognosis of patients; furthermore, blood counts and bone marrow recovery kinetics following chemotherapy are associated with the prognosis of AML. Yanada et al. demonstrated that a PLT count of > 320 × 10^9^/L at CR (75th percentile of their cohort) was significantly associated with better RFS [9]. Umit reported that a PLT count of > 525 × 10^9^/L is an independent prognostic factor in AML patients [10].

Although important discoveries between PLT counts and AML prognosis were revealed in these studies, some limitations were observed. All of these studies used a single measurement, ignoring the dynamic change over time [11]. PLT counts vary over time after induction chemotherapy until bone marrow recovery. Pretreatment and posttreatment data of AML patients are necessary to complement the prognosis evaluation at the same time. Single measurements may preclude the examination of long-term trajectories of PLT counts and their relevance to AML development. PLT count variability may predict the response to chemotherapy and can be used to assess the prognosis. A life course approach using multiple PLT count measurements over time after induction chemotherapy may shed new light on PLT count trajectories and their relevance to the AML prognosis [12]. In this study, we initially used a group-based trajectory model (GBTM) to identify latent PLT count trajectory groups and investigated the association between trajectory group membership and all-cause mortality in AML. GBTM has long been used in psychology and criminology research, but is now increasingly used in clinical research [13]. GBTM assumes that the baseline patient population is heterogeneous and consists of individuals with a similar disease course. It classifies individuals into groups (or trajectories) based on how they develop over time and can distinguish the defining characteristics of their members [14, 15]. Such a method will likely provide critical information for identifying people with an increased risk of death from AML and has implications for timely intervention.

## Methods

### Patients and treatment

Data of 149 newly diagnosed AML patients between May 1, 2013 and May 1, 2021 admitted in Qianfoshan Hospital affiliated with Shandong University were evaluated retrospectively, and all patients were followed up until July 1, 2021. Diagnosis was made based on the 2006 World Health Organization classification. All studied patients with AML received induction/reinduction chemotherapy and other diagnostic/therapeutic standard clinical interventions if there was an absolute clinical indication at the given disease state(s) [10]. PLT transfusion was administered in patients with active hemorrhage and PLT counts of < 20 × 10^9^/L. Platelet-stimulating agents were provided in patients with a PLT counts of < 30 × 10^9^/L and who stopped taking medications when the PLT counts was > 80 × 10^9^/L. The patients aged > 16 years; who received standard induction chemotherapy: (1) DA (daunorubicin [DNR] 40–60 mg/m^2^/day by intravenous infusion from days 1 to 3 and cytarabine [Ara-C] 100–200 mg/m^2^/day by intravenous infusion from days 1 to 7), (2) IDA (idarubicin [IDA] 8–12 mg/m^2^/day by intravenous infusion from days 1 to 3 and Ara-C 100–200 mg/m^2^/day by intravenous infusion from days 1 to 7), (3) MA (mitoxantrone 8 mg/m^2^/day by intravenous infusion from days 1 to –3 and Ara-C 100–200 mg/m^2^/day by intravenous infusion from days1 to 7), or (4) HA (homoharringtonine 2-2.5 mg/m^2^/day by intravenous infusion from days 1 to 3 and Ara-C 100–200 mg/m^2^/day by intravenous infusion from days 1 to 7); and whose PLT count was measured from the first day of the first cycle of induction therapy until 40 days and was measured every 2–3 days during the period of chemotherapy were included in the study. Patients with PLT measured  < 8 times, diagnosed with acute promyelocytic leukemia and secondary (therapy-associated or evolving from previous hematologic conditions) AML, and who received palliative treatment but did not meet our chemotherapy dose range were excluded.

CR was defined as < 5% blasts in the bone marrow, absence of extramedullary disease, peripheral neutrophil count of ≥ 1 × 10^9^/L, PLT count of ≥ 100 × 10^9^/L, and independence from red cell transfusions. CR_1_ was defined as achieving CR after the first cycle of induction chemotherapy. Follow-up times were measured from the last recorded PLT count to the date of death from any cause or the last follow-up. The mortality rate per 1,000 person-days referred to the number of deaths per 1,000 person-days of follow-up.

The baseline characteristics, including age, gender, white blood cell (WBC) count, hemoglobin (Hb) level, PLT count, and bone marrow blasts at the time of diagnosis, were collected. The study was approved by the ethics committee of Qianfoshan Hospital affiliated with Shandong University and was conducted in accordance with the ethical standards of the World Medical Association Declaration of Helsinki.

### Statistical analysis

The characteristics across different PLT count groups were compared using statistical analysis. Normality and homogeneity of variance tests were performed to analyze the continuous data. Analysis of variance was used to compare the differences between groups of independent variables that met the conditions. The Kruskal–Wallis nonparametric test was used to compare the differences between groups that did not meet the normality or homogeneity of variance. For categorical variables, if the theoretical frequency of the four tables was greater than or equal to 5, the differences between groups were compared using χ^2^ test. If the theoretical frequency was less than 5 and greater than or equal to 1, the differences between the groups were compared using the Fisher’s exact test. Poisson regression analysis was used to compare the death density data between the groups. The PROC TRAJ procedure (SAS 9.4) was used to perform GBTM in order to identify the PLT count trajectories over time. One trajectory was fitted using the censored normal model with a linear, quadratic, and cubic trajectory function of days, and then the number of groups was changed from 1 to 5. Each time the trajectory was fitted, the GBTM displayed the posterior probability that trajectory members belong to each group. We selected models with the smallest absolute Bayesian information criterion (BIC) and ensured that the average posterior probability of each trajectory group was not less than 70% and that the members of each group were not less than 5%.

To study the association between diverse trajectories of PLT counts and survival outcomes, the Kaplan–Meier survival curve of each PLT trajectory group was plotted. The log-rank test was used to determine whether statistical differences exist in all-cause mortality among the groups. The Cox proportional hazard model was used to evaluate the association between PLT count trajectories and mortality risk, with gradual adjustment for baseline age, gender, WBC counts, and bone marrow blasts.

Finally, to test the robustness of the risk estimates, an analysis was performed using a down-sampling approach. This approach performs trajectory analysis using a randomly selected sample of 147, 145, …, 101 individuals and then repeats the above analysis with the same trajectory parameters and number, thus verifying the repeatability of the trajectory and the stability of the PLT count trajectory effect.

The R 4.0.2 and SAS 9.4 were used to perform all data analyses, and a two-sided *p* value of less than 0.05 was considered significant.

## Results

### Baseline clinical characteristics

**Fig. 1:**
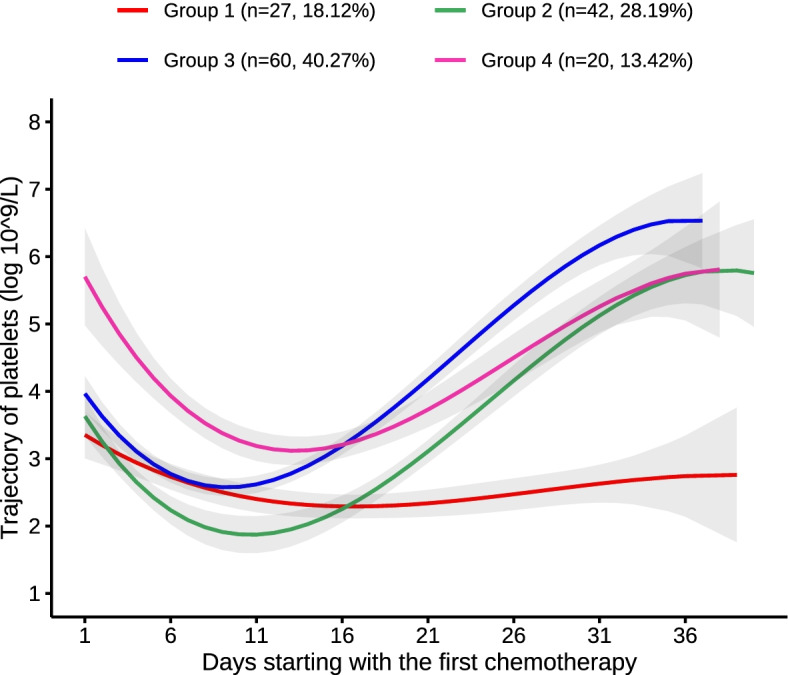
Trajectory of PLT counts after induction chemotherapy over time. Each trajectory is represented by a different color. group1: low-stability group, group2: low-level decrease-medium elevation group, group3: low-level decrease-high level elevation group, group4: high-level decrease-medium elevation group

This retrospective study included 149 AML patients. The four trajectory curves are shown in Fig. 1. The PLT count trajectories included group 1 (low- stability group: *n* = 27, 18.12%), group 2 (low-level decrease–medium elevation group: 42, 28.19%), group 3 (low-level decrease–high elevation group: 60, 40.27%), and group 4 (high-level decrease–medium elevation group: 20, 13.42%). In the low-stability group, the PLT counts remained between 2.29 and 3.35. In the low-level decrease–medium elevation group, the PLT counts declined from 3.63 to 1.87 and increased to 5.80. In the low-level decrease–high elevation group, the PLT counts declined from 3.97 to 2.58 and increased rapidly to 6.53. In the high-level decrease–medium elevation group, the PLT counts declined from 5.70 to 3.12 and increased to 5.81.

Tables 1 and 2 present the model fitting of the GBTM results; four different types of PLT count trajectories had the best performance in terms of BIC, with an average posterior probability of ≥ 70%. The development trajectory of PLT counts was evaluated, which showed polynomial orders of 3, 3, 3, and 3, respectively. The detailed parameter estimations for these four trajectories are presented in Table 1. The mean posterior probabilities for these four trajectory groups were 91.02%, 85.58%, 92.05%, and 86.78%, respectively.

**Table 1:** The model fitting of optimal trajectories of platelet counts

Number of groups	Variables	Highest order of trajectory curve	BIC	% Participants	Mean posterior probabilities
1	Platelets	3	-2798.27	100%	100%
2	Platelets	3/3	-2634.87	26.85%73.15%	92.83%/96.21%
3	Platelets	3/3/3	-2588.60	25.50%/8.72%/65.77%	91.18%/96.33%/95.50%
4	**Platelets**	**3/3/3/3**	**-2562.97**	**18.12%/28.19%/ 40.27%/13.42%**	**91.02%/85.58%/ 92.05%/86.78%**
5	Platelets	3/3/3/3/3	-2528.54	1.34%/26.17%/8.72%/ 52.35%/11.41%	100.00%/93.29%/95.48%/ 93.98%/91.04%

*BIC* Bayesian information criterion; the optimal model is highlighted in bold

**Table 2:** Parameter estimates for the optimal group-based trajectory model

Variables	Group	Coefficient of trajectory equations			
		Intercept	Linear	Quadratic	Cubic
Platelets	1	3.5174	-0.1721	0.0074	-0.0001
	2	4.0584	-0.4547	0.0273	-0.0004
	3	4.3558	-0.4181	0.0282	-0.0004
	4	6.2008	-0.5253	0.0266	-0.0003

Group 1 to group 4 indicate different trajectories of platelet counts

Table 3 presents the baseline characteristics according to the different trajectories of PLT counts. The AML mortality rates per 1,000 person-days were 1.90, 0.58, 0.47, and 0.70 per 1,000 person-years, respectively. Significant differences in HB, PLT, follow-up time, and CR were observed (*p* < 0.01). Compared with the other groups, group 4 had lower WBC counts and percentage of bone marrow blasts and higher values of HB, whereas group 4 had a lower CR_1_.

**Table 3:** Baseline characteristics according to different trajectories of platelets

Characteristics	Group 1(*N* = 27)	Group 2(*N* = 42)	Group 3(*N* = 60)	Group 4(*N* = 20)	*P*
The number of deaths, n	15	15	8	5	
Mortality rate per 1000 person-days	1.90	0.58	0.47	0.70	0.004
Follow-up times, (days)	99.00 (43.00, 289.50)	355.50 (182.25, 814.00)	327.50 (156.75, 1055.00)	185.50 (129.75, 464.25)	0.002
Age, year	54.00 (45.50, 64.00)	53.00 (43.00, 60.00)	47.50 (34.00, 57.75)	52.00 (43.25, 65.00)	0.209
Female, n (%)	14 (51.85)	19 (45.24)	33 (55.00)	8 (40.00)	0.612
WBC counts, 10^9^/L	7.07 (1.50, 43.52)	17.05 (6.59, 46.22)	13.77 (6.78, 54.94)	3.57 (2.09, 16.16)	0.083
HB, g/L	69.11 ± 19.11	71.52 ± 20.27	86.67 ± 23.88	82.30 ± 26.81	< 0.001
Bone Marrow Blasts(%)	0.60 (0.38, 0.79)	0.57 (0.37, 0.87)	0.60 (0.42, 0.80)	0.47 (0.40, 0.75)	0.877
Missing sample	0	1	9	2	
PLT, 10^9^/L	34.00 (17.50, 56.50)	20.50 (10.25, 36.25)	41.00 (20.75, 74.25)	149.00 (120.00, 232.00)	< 0.001
CR_1_, n(%)	0 (0)	30 (71.43)	50 (83.33)	5 (25.00)	< 0.001

The data were presented as mean ± SDs, median (P_25_, P_75_), number, or number (%). *WBC* white blood cell, *HB* hemoglobin, *PLT* platelet, Group 1:low-stability group; Group 2: low-level decrease-medium elevation group; Group 3: low-level decrease-high level elevation group; Group 4: high-level decrease-medium elevation group

^*^ *p* < 0.001; † *p* < 0.01; ‡ *p* < 0.05

**Fig. 2:**
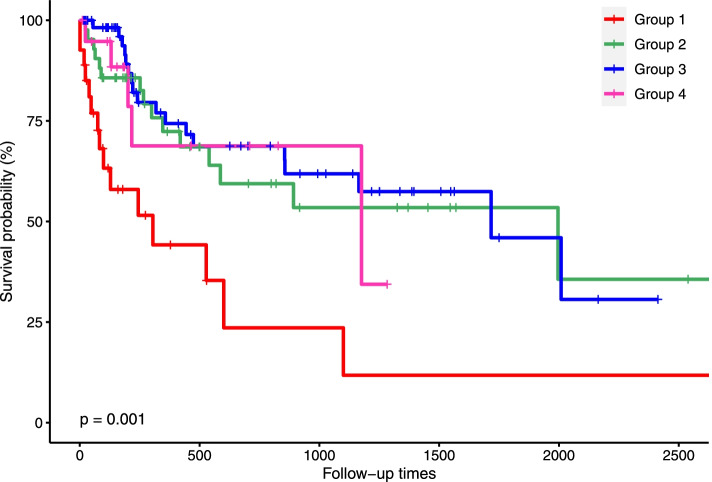
Kaplan–Meier survival curve of PLT count trajectories after induction chemotherapy

Results of the Kaplan–Meier survival curve analysis are shown in Fig. 2. A log-rank test was performed, which showed a significant difference in the survival rates between the different trajectory groups. Table 4 presents the association between trajectory group membership and AML, using various Cox proportional hazard models. With group 1 as the reference group, the hazard ratios (HRs) for group 2, group 3, and group 4 were 0.32 (95% confidence interval (CI), 0.15–0.68), 0.31 (95% CI, 0.15–0.63), and 0.35 (95% CI, 0.13–0.89) after adjusting for age and gender. With group 1 as the reference group, the HRs for group 2 and group 3 were 0.33 (95% CI, 0.14–0.77) and 0.31 (95% CI, 0.14–0.67) after adjustment for age, gender, WBC counts and bone marrow blasts. After adjusting for other potential clinical characteristics, including age, gender, WBC counts, bone marrow blasts, and PLT counts, group 2 and group 3 were still positively associated with an AML mortality risk; the HRs for group 2 and group 3 were 0.35 (95% CI, 0.15–0.81) and 0.30 (95% CI, 0.14–0.66) respectively.

**Table 4:** HRs and 95% CIs of trajectories on mortality risk

	Model^a^	Model^b^	Model^c^	Model^d^
Trajectories groups				
Group 1	Reference	Reference	Reference	Reference
Group 2	0.34 (0.16–0.74)†	0.32 (0.15–0.68)†	0.33 (0.14–0.77)‡	0.35 (0.15–0.81)‡
Group 3	0.29 (0.14–0.59)*	0.31 (0.15–0.63)†	0.31 (0.14–0.67)†	0.30 (0.14–0.66)†
Group 4	0.36 (0.13–0.99)‡	0.35 (0.13–0.89)‡	0.41 (0.15–1.09)	0.27 (0.07–1.09)
age		1.03 (1.01–1.05)†	1.03 (1.01–1.05)†	1.03 (1.01–1.05)†
gender		0.81 (0.46–1.42)	0.68 (0.35–1.29)	0.65 (0.33–1.26)
WBC			1.00 (0.99–1.01)	1.00 (0.99–1.01)
Bone marrow blasts			2.79 (0.64–12.09)	3.06 (0.68–13.74)
PLT				1.00 (0.99–1.01)

HRs, hazard ratios; CIs, confidence intervals; group 1 to group 4 indicate different trajectories of platelets

^a^Adjusting for platelet trajectories

^b^Adjusting for platelet trajectories, age, gender

^c^Adjusting for platelet trajectories, age, gender, WBC, and bone marrow blasts (bone marrow blasts were damaged, causing 12 to be damaged, leaving 137 people in the model, of whom 87 survived and 50 died)

^d^Adjusting for platelet trajectories, age, gender, WBC count, bone marrow blasts (bone marrow blasts were damaged, causing 12 to be damaged, leaving 137 people in the model, of whom 87 survived and 50 died.), and PLT

^*^ *p* < 0.001; † *p* < 0.01; ‡ *p* < 0.05

**Fig. 3:**
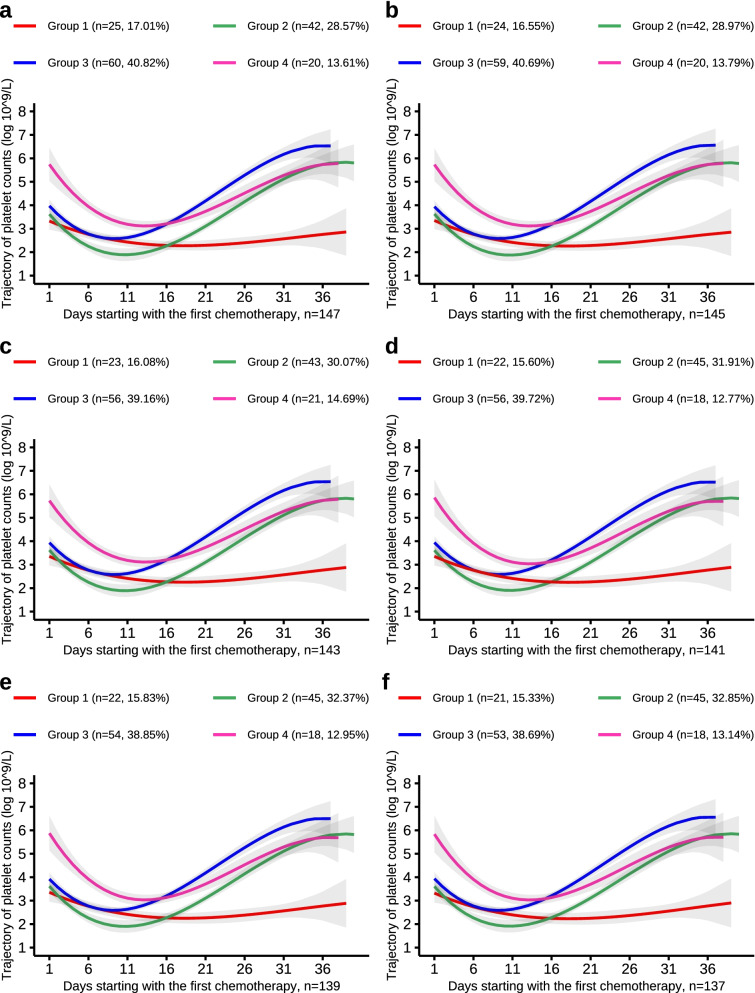
PLT trajectories of the down-sample of number from 147 to 137

**Fig. 4:**
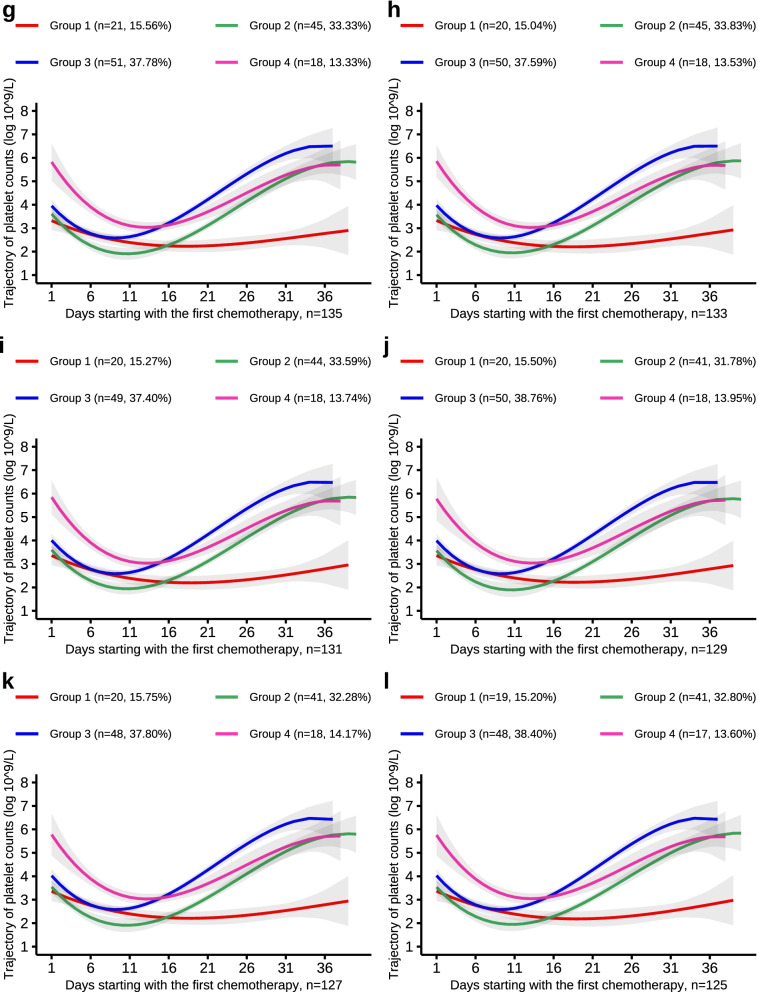
PLT trajectories of the down-sample of number from 135 to 125

**Fig. 5:**
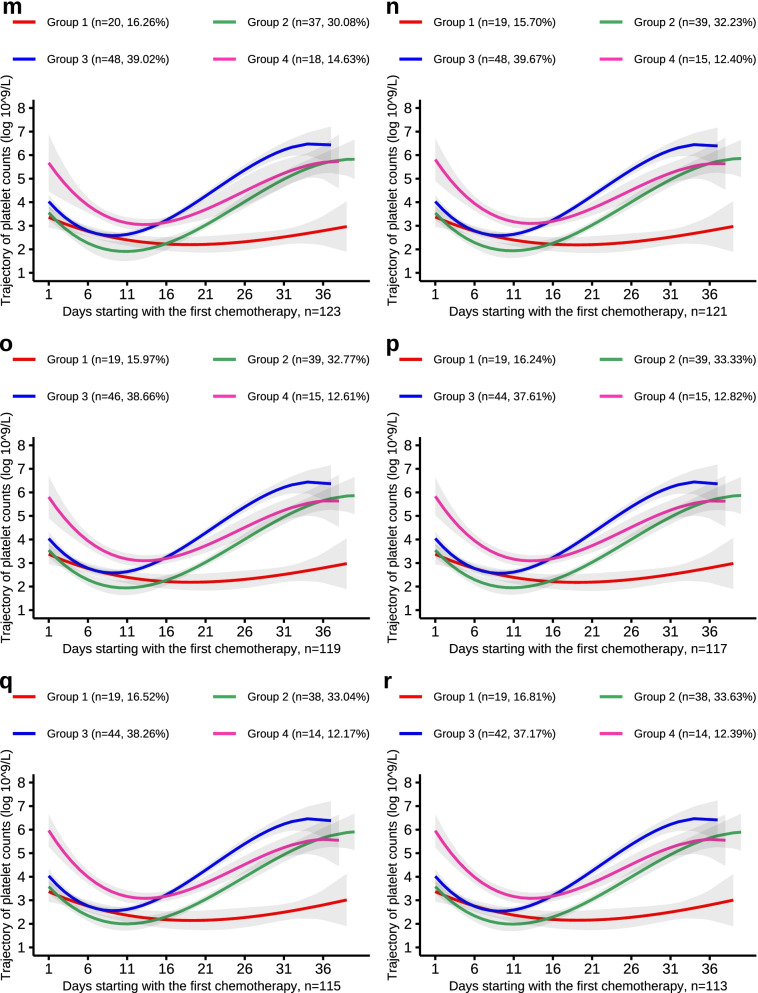
PLT trajectories of the down-sample of number from 123 to 113

**Fig. 6:**
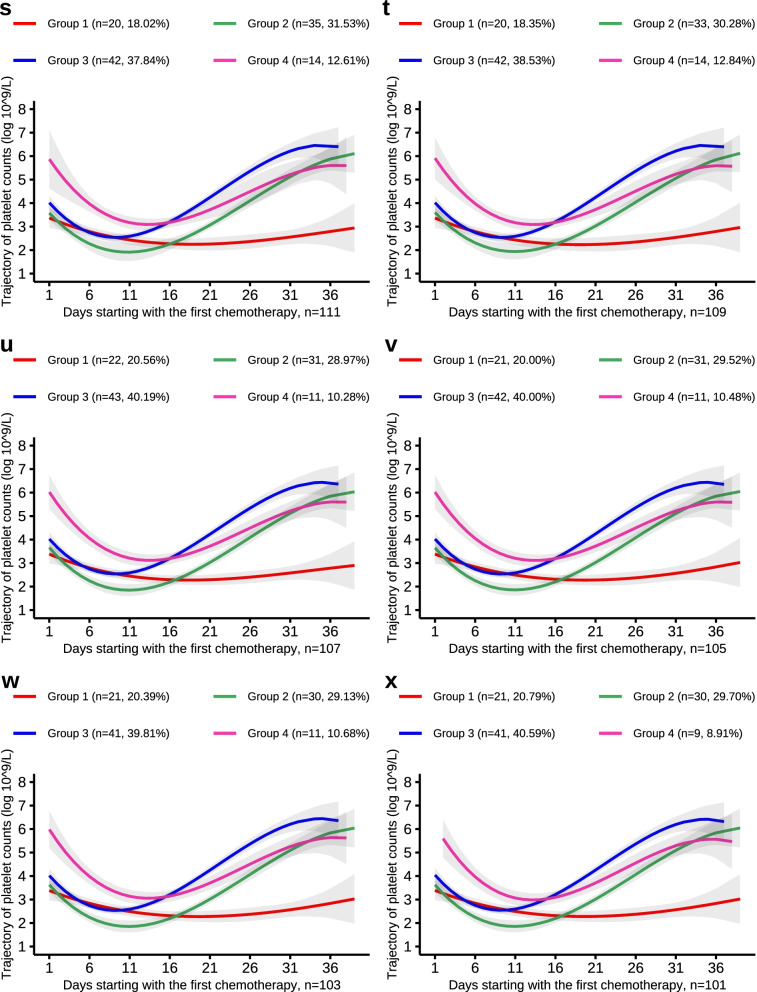
PLT trajectories of the down-sample of number from 111 to 101

The aggregated results are shown in Figs. 3,4,5,6 and the trajectory curves showed similar results for the overall population. The probability of reassignment to the original trajectory (Table 5) was relatively high.

**Table 5:** The probability of being reassigned to the original trajectory*

Number	Group 1	Group 2	Group 3		Group 4	
149	Reference					
147	(25/25) 100%	(42/42) 100%		(60/60) 100%		(20/20) 100%
145	(24/24) 100%	(42/42) 100%		(59/59) 100%		(20/20) 100%
143	(23/23) 100%	(42/42) 100%		(56/58) 96.55%		(20/20) 100%
141	(22/23) 95.65%	(42/42) 100%		(56/58) 96.55%		(17/18) 96.44%
139	(22/23) 95.65%	(42/42) 100%		(54/56) 96.43%		(17/18) 96.44%
137	(21/22) 95.45%	(42/42) 100%		(53/55) 96.36%		(17/18) 96.44%
135	(21/22) 95.45%	(42/42) 100%		(51/53) 96.23%		(17/18) 96.44%
133	(20/22) 90.91%	(41/41) 100%		(50/52) 96.15%		(17/18) 96.44%
131	(20/21) 95.24%	(41/41) 100%		(49/51) 96.08%		(17/18) 96.44%
129	(20/21) 95.24%	(39/39) 100%		(50/51) 98.04%		(17/18) 96.44%
127	(20/21) 95.24%	(39/39) 100%		(48/49) 97.96%		(17/18) 96.44%
125	(19/21) 90.48%	(38/38) 100%		(48/49) 97.96%		(16/17) 94.12%
123	(20/21) 95.24%	(36/36) 100%		(48/49) 97.96%		(17/17) 100%
121	(19/21) 90.48%	(36/36) 100%		(48/48) 100%		(15/16) 93.75%
119	(19/21) 90.48%	(36/36) 100%		(46/46) 100%		(15/16) 93.75%
117	(19/21) 90.48%	(36/36) 100%		(44/44) 100%		(15/16) 93.75%
115	(19/21) 90.48%	(35/35) 100%		(44/44) 100%		(14/15) 93.33%
113	(19/21) 90.48%	(35/35) 100%		(42/42) 100%		(14/15) 93.33%
111	(20/21) 95.24%	(33/33) 100%		(42/42) 100%		(14/15) 93.33%
109	(20/21) 95.24%	(31/31) 100%		(42/42) 100%		(14/15) 93.33%
107	(21/21) 100%	(31/31) 100%		(42/42) 100%		(11/13) 84.62%
105	(20/20) 100%	(31/31) 100%		(41/41) 100%		(11/13) 84.62%
103	(20/20) 100%	(30/30) 100%		(41/41) 100%		(11/12) 91.67%
101	(20/20) 100%	(30/30) 100%		(40/40) 100%		(9/11) 81.82%

^*^Taking the trajectory results of 147 patients as an example, 25 patients in the trajectory model of 149 people were assigned to group 1, and these 25 people were reassigned to group 1 in the trajectory model of 147 people. The probability of being reassigned to the original trajectory was 100%

## Discussion

In this retrospective study, we identified four distinct trajectories of PLT counts and disclosed the association between these trajectories and the prognosis of newly diagnosed AML in patients treated with standard chemotherapy. To our knowledge, this study was the first to report the dynamic trajectories of PLT counts after the first cycle of induction chemotherapy in patients with AML.

Among the four trajectories of PLT counts over time, the low-level decrease–medium elevation group and low-level decrease–high elevation group were followed up and showed a higher complete recovery rate after the first cycle of induction chemotherapy. Early CR achievement is associated with longer overall survival and better quality of life in patients with AML [6]. For example, Megan Othus et al. suggested that early remission (after the first induction cycle) has better survival outcomes than those who required two cycles of chemotherapy in order to achieve CR, even after adjustment for other risk factors [16]. Undoubtedly, patients with a PLT count of less than 30 are at risk of severe bleeding. Thrombocytopenia is a common problem in leukemia and can lead to hemorrhagic complications [17]. Patients are at an increased risk of bleeding owing to chemotherapy-related thrombocytopenia. PLT counts declined and did not increase between 15 and 20 days in group 1, indicating a low likelihood of recovery at later stages and higher mortality. This finding is consistent with the clinical practice. Noticeably, group 1 had the lowest PLT counts on the first day of the first cycle of induction therapy and had the least rapid fall of PLT counts within approximately 6 days, which may be a novel marker for poor prognosis. Timely interventions, such as hematopoietic stem cell transplantation, may be considered in this group of patients.

Dimitrios indicated that group 3 (pretreatment PLT counts of > 130 × 10^9^/L) had the worst prognosis compared with group 1 (pretreatment PLT count of < 25 × 10^9^/L) and group 2 (pretreatment PLT counts 25–130 × 10^9^/L); group 3 had favorable prognostic features, high levels of endogenous thrombopoietin (TPO), and high expression of TPO receptor (C-MPL), but with low chemotherapy response and poor prognosis [18]. The TPO/MPL regulatory pathway plays a critical role in the interaction of human leukemic stem cells (LSCs) with the hematopoietic microenvironment; the upregulation of the TPO/c-MPL signaling pathway may protect the LSCs from the effects of chemotherapy as treatment for AML and is associated with chemoresistance and AML recurrence [19, 20]. Similar to the Dimitrios’ study, group 4 had either normal or elevated PLT counts, marked by lower WBC counts, a lower percentage of bone marrow blasts, and higher HB level. After the first cycle of induction chemotherapy, the CR rate was 5%. Dimitrios only measured the PLT counts at therapy initiation in patients with AML, but failed to determine the PLT counts after induction chemotherapy and its association with pre-treatment. However, our study described the PLT count dynamics and found that PLT counts did not significantly increase after the first cycle of induction chemotherapy in this special group of patients. Patients with low CR_1_ may require novel chemotherapy to achieve CR as soon as possible and more frequent follow-up testing to consolidate treatment with curative intent. However, this finding should be verified in a prospective randomized controlled trial [21]. Unfortunately, the dynamic changes in TPO and MPL in group 4 were not measured before and after chemotherapy. Group 4 was a heterogeneous group with lower WBC counts, a lower percentage of bone marrow blasts, higher HB level, and lower CR rate. The molecular mechanism associated with this heterogeneity and its role in disease progression require further study.

PLT have multiple functions, including hemostasis, tissue remodeling after injury, wound healing, and participation in inflammatory and immune responses [22]. However, they also play an important role in the disease prognosis. The association between PLT count trajectories and outcomes has been reported in several diseases, including severe coronavirus disease 2019 [23], liver fibrosis [24], and severe burns [25]. However, the novel finding was that the dynamic trajectory of PLT count was a significant predictor of all-cause mortality in patients with acute myeloid leukemia.

Pre-treatment information with cytogenetics and various genetic abnormalities from patients is undoubtedly the basis for selecting the proper treatment method, whereas post-treatment information can be regarded as a “true response” rather than a “predictive factor” [3]. Therefore, pretreatment and post-treatment information should be carefully considered [3]. Our study demonstrates the significance of the GBTM in identifying hidden information in repeated measurements of PLT counts. The primary advantage of GBTM is that it does not assume a priori the existence of trajectories of a particular form, but allows unique latent developmental trajectories that can be learned from the data [12]. Models that include trajectory information will have better predictions than those using only single PLT measurements. As PLT counts are extremely easy to detect, they may be regarded as a new easy-to-use method for exploring the prognostic value of multiple PLT measurements in countries with limited laboratory facilities [10].

Limitations of our findings also need to be recognized. It was a retrospective study with a limited sample of 149 patients. However, GBTM and consecutive data collection were able to identify statistically significant findings in the sample [26–28]. In addition, because various mutations were partly deficient, we could not determine the different molecular and cytogenetic variations among the trajectories of PLT counts. The mechanisms responsible for the differences in PLT count trajectories require further investigation. Although our statistical methods were limited, they were particularly suited for the dynamic analysis of continuous data and generated novel findings [28]. Employing similar data collection and statistical methods with larger sample sizes, such as those included in multicenter prospective clinical studies, would help confirm our findings and identify additional relevant trends.

## Conclusion

In summary, we identified four distinct trajectories of PLT counts associated with all-cause mortality after the first cycle of induction chemotherapy in patients with AML. This study provides new insights into the prognostic significance of multiple PLT counts.

## Data Availability

The datasets generated and/or analysed during the current study are not publicly available due to information that could compromise research participant privacy, but are available from the corresponding author on reasonable request.

## Abbreviations

AML: Acute myeloid leukemia
CR: Complete remission
PLT: Platelet
WBC: White blood cell
Cri: Complete remission with incomplete blood count recovery
CRp: Complete remission with platelet counts < 100 × 10^9^/L
GBTM: Group-based trajectory model
HB: Hemoglobin
BIC: Bayesian information criterion
HRs: Hazard ratios
Cis: Confidence intervals
LSCs: Leukemic stem cells
TPO: Thrombopoietin
C-MPL: TPO receptor
