# Correction: Dynamic trajectory of platelet counts after the first cycle of induction chemotherapy in AML patients

**DOI:** 10.1186/s12885-022-09715-w

**Published:** 2022-06-13

**Authors:** Yazhen Bi, Zhaohui Wang, Saran Feng, Yan Wang, Yang Zhao, Hong Li, Jingyi Yu, Qian Liu, Chuansheng Zhu, Mingzhuo Li

**Affiliations:** 1 Shandong Qianfoshan Hospital, Cheeloo College of Medicine, Shandong University, Jinan, Shandong China; 2 Shandong Provincial Qianfoshan Hospital, Shandong University, Jinan, Shandong China; 3 Department of Hematology, Haici Medical Group Qingdao, Qingdao, Shandong China; 4 Department of Biostatistics, School of Public Health, Nanjing Medical University, Nanjing, Jiangsu China; 5 Center for Big Data Research in Health and Medicine, The First Affiliated Hospital of Shandong First Medical University & Shandong Provincial Qianfoshan Hospital, Jinan, Shandong China

**Correction: BMC Cancer 22, 477 (2022)**

**https://doi.org/10.1186/s12885-022-09601-5**

Following publication of the original article [1], the authors identified the following errors in the article:

1. The color in Fig. 1 was incorrectly marked and has been updated in this correction article.
2. In Table 4, the platelet variables (PLT) in the penultimate line should be in the last line.

The original article [1] has been corrected.

**Fig. 1:**
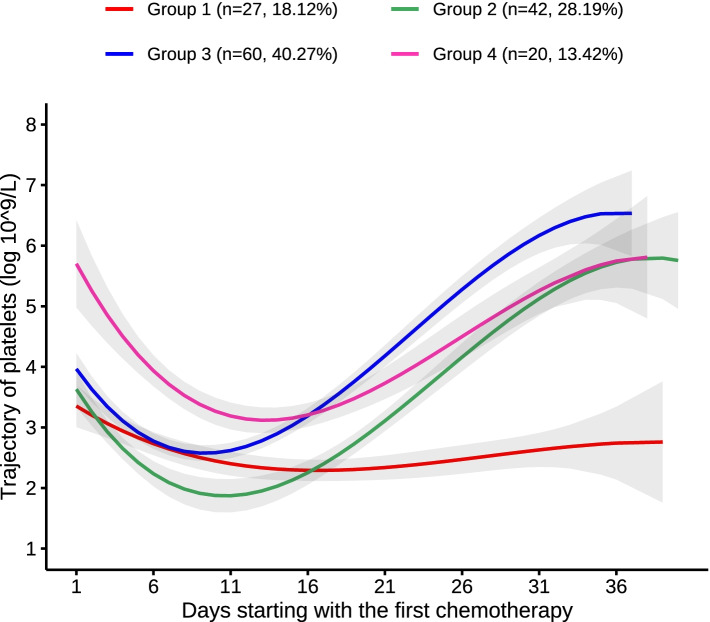
Trajectory of PLT counts after induction chemotherapy over time. Each trajectory is represented by a different color. group1: low-stability group, group2: low-level decrease-medium elevation group, group3: low-level decrease-high level elevation group, group4: high-level decrease-medium elevation group

**Table 4:** HRs and 95% CIs of trajectories on mortality risk

	Model^a^	Model^b^	Model^c^	Model^d^
Trajectories groups				
Group 1	Reference	Reference	Reference	Reference
Group 2	0.34 (0.16–0.74)†	0.32 (0.15–0.68)†	0.33 (0.14–0.77)‡	0.35 (0.15–0.81)‡
Group 3	0.29 (0.14–0.59)*	0.31 (0.15–0.63)†	0.31 (0.14–0.67)†	0.30 (0.14–0.66)†
Group 4	0.36 (0.13–0.99)‡	0.35 (0.13–0.89)‡	0.41 (0.15–1.09)	0.27 (0.07–1.09)
age		1.03 (1.01–1.05)†	1.03 (1.01–1.05)†	1.03 (1.01–1.05)†
gender		0.81 (0.46–1.42)	0.68 (0.35–1.29)	0.65 (0.33–1.26)
WBC			1.00 (0.99–1.01)	1.00 (0.99–1.01)
Bone marrow blasts			2.79 (0.64–12.09)	3.06 (0.68–13.74)
PLT				1.00 (0.99–1.01)

*HRs* hazard ratios, *CIs* confidence intervals; group 1 to group 4 indicate different trajectories of platelets

^a^Adjusting for platelet trajectories

^b^Adjusting for platelet trajectories, age, gender

^c^Adjusting for platelet trajectories, age, gender, WBC, and bone marrow blasts (bone marrow blasts were damaged, causing 12 to be damaged, leaving 137 people in the model, of whom 87 survived and 50 died)

^d^Adjusting for platelet trajectories, age, gender, WBC count, bone marrow blasts (bone marrow blasts were damaged, causing 12 to be damaged, leaving 137 people in the model, of whom 87 survived and 50 died.), and PLT

* *p*<0.001; † *p*<0.01; ‡ *p*<0.05
